# Foreign Body in Paranasal Sinus: A Late Clinical Presentation

**DOI:** 10.1155/2019/4386938

**Published:** 2019-01-06

**Authors:** Francisco Monteiro, Pedro Oliveira, Artur Condé

**Affiliations:** Department of Otorhinolaryngology, Head and Neck Surgery, Hospital Center of Vila Nova de Gaia/Espinho, Rua Conceição Fernandes, S/N, 4434-502 Vila Nova de Gaia, Portugal

## Abstract

The occurrence of foreign bodies in paranasal sinuses is extremely rare. The symptoms are vague and usually discovered after extra/intracranial complications. They may, therefore, go unnoticed if there isn't a strong clinical suspicion. We present a clinical case of a 64-year-old woman with a paranasal sinus microsurgery history more than 30 years ago, who presented with headache and purulent rhinorrhea. A glass tubular structure was discovered in the ethmoid complex. She underwent functional endoscopic sinus surgery. Since glass is an inert material that doesn't cause foreign body reaction, the patient may not present any symptom or sign. However, if there is obstruction in the drainage of the ostiomeatal complex, it can manifest itself as rhinosinusitis, which we believe happened in our case. To the best of our knowledge, this is probably the first reported case of a glass removed from the ethmoidal sinuses, diagnosed with more than 30 years of evolution.

## 1. Introduction

The occurrence of foreign bodies in paranasal sinuses is extremely rare, and approximately 70% of these cases are associated with some form of maxillofacial trauma, while others are related to surgical treatments after dental problems [1]. Due to improvement of radiology imaging techniques in the last decades, late presentation of foreign bodies in the frontal sinuses are rare [2]. Symptoms are vague and usually discovered after extracranial and intracranial complications or by occasional radiology images. They may, therefore, go unnoticed if there isn't a strong clinical suspicion [1].

Thus, the authors present a case of a rhinolith in the ethmoidal sinuses, with more than 30 years of evolution.

## 2. Case Presentation

The authors present a clinical case of a 64-year-old female patient, with no relevant medical history (including trauma), who went to the emergency department of our hospital presenting a clinical scenario with a few years of left intense headache and left hemifacial pain that worsen with head movements, purulent and sometimes greenish rhinorrhea, and cacosmia.

The patient had a history of microsurgery of paranasal sinus more than 30 years ago in another hospital, due to complaints compatible with chronic rhinosinusitis.

The otorhinolaryngologic examination, in particular anterior rhinoscopy complemented with nasofibroscopy, revealed a congested nasal mucosa, a dark friable lesion at the middle meatus, and the first diagnostic hypotheses were rhinolith/fungal rhinosinusitis. In the emergency department, she underwent a cranial computed tomography (CT) scan that identified “… lesion at the level of the left paranasal sinuses compatible with fungal rhinosinusitis.”

**Figure 1:**
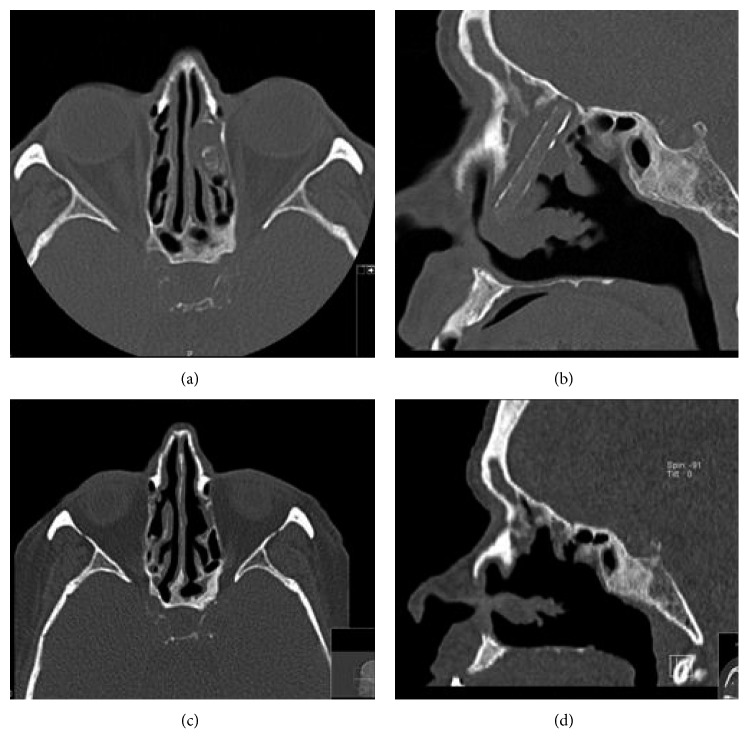
(a, b) Axial and sagittal cuts of a paranasal CT scan showing cylindrical foreign body, in the left nasal fossa and ethmoid sinus; (c, d) coronal and sagittal CT scans 6 months after FESS, with removal of the foreign body.

She was referred to an Ear, Nose, and Throat appointment. A paranasal sinus CT scan identified “… tubular foreign body, about 37 × 5 mm prehypertensive, filled with soft tissues inside the left nasal fossa, the left middle meatus and anterior ethmoid. Also, signs of chronic inflammation were observed, with thickening of the right frontal sinus mucosa, the ethmoidal cells, the sphenoid and the maxillary sinuses, associated with sclerotic osteitis of the bone walls that delimit the maxillary and sphenoidal sinuses” (Figure 1).

She was also subjected to a complementary study with magnetic resonance of the SPN to best characterize the lesion which revealed “… centred on the left middle meatus, but extending to the posterior portion of the complex anterior ethmoid on the same side, where there is apparently a focal bone continuity solution of the base of the anterior floor, a cylindrical/tubular configuration void with peripheral soft tissue component. It is about 3.8 cm larger in diameter and probably corresponds to extrinsic material and, given its regular configuration, it is unlikely that the alternative hypothesis is a mycetoma.”

She underwent functional endoscopic sinus surgery (FESS) with antrostomy, anterior and posterior ethmoidectomy, debridement of the necrotic tissue and removal of the foreign body that proved to be a cylindrical structure, which was a piece of glass. During the follow-up, in 6 months after surgery, the patient remained completely asymptomatic and performed another CT scan which showed no signs of inflammation.

## 3. Discussion

With the recent best availability of computerized tomography and magnetic resonance, it is now unlikely that foreign bodies in paranasal sinuses go unnoticed. Since glass is an inert material that does not cause foreign body reaction, the patient may not present any complaint or symptom unlike other materials that can generate osteomyelitis, frontal sinusitis, or CSF fistula [3, 4]. In our patient, the glass structure was probably left unnoticed in the previous microsurgery or in the follow-up and probably didn't cause a foreign body reaction. However, such in this case, if there is obstruction in the nasofrontal duct or in drainage of the ostiomeatal complex, it may manifest itself with symptoms of rhinosinusitis. It is important to keep in mind that more than 50% of the foreign bodies in the paranasal sinuses are found in the maxillary sinuses [1]. To the best of our knowledge, this is probably the first reported case of a glass removed from the ethmoidal sinuses, diagnosed with more than 30 years of evolution.

## 4. Conclusion

This case warns us of the need and importance of always maintaining a high degree of clinical suspicion so as not to devalue some symptoms that, appearing benign, could lead to severe complications, both extracranial and intracranial.
